# Attitude of Veterinarians Toward Self-Informed Animal Owners Affects Shared Decision Making

**DOI:** 10.3389/fvets.2021.692452

**Published:** 2021-10-20

**Authors:** Roswitha Merle, Alina M. Küper

**Affiliations:** Department of Veterinary Medicine, Institute for Veterinary Epidemiology and Biostatistics, Freie Universität Berlin, Berlin, Germany

**Keywords:** relationship-centered care, veterinary animal-owner communication, veterinary medicine, partnership building, empathy, shared decision making

## Abstract

The aims of this study were to investigate the role of the veterinarian characteristics (e.g., age, gender, self-estimation, use of the internet), and their attitudes concerning animal owners seeking self-information. A particular focus was laid on any association between shared decision making (SDM), age and gender. In an online survey, 527 German veterinarians were asked about their attitude regarding SDM principles and their experiences with self-informed animal owners. The factors associated with veterinarians' perception of SDM were investigated in a multivariable linear regression model. A recently published structural equation model consolidated the application of SDM, empathic behavior, and veterinarians' evaluation of self-education as latent factors. Interconnected questionnaire items were processed using an exploratory factor analysis to 11 interpretable factors. Veterinarians who assumed therapy failure was associated with themselves had significantly higher rates of SDM (*p* = 0.002). In contrast, SDM was significantly lower (*p* = 0.002) if they assumed that therapy failure was due to the animal's owners. SDM was negatively associated with the perceived quality of the pet owners' self-information (*p* < 0.001) and if skepticism was perceived as the reason for seeking the self-information (*p* = 0.001). Veterinarians who advised against self-information (*p* = 0.006) and those who assumed that self-information of animal owners goes along with uncertainty (*p* = 0.001) had low SDM values (*p* = 0.006). Asking the animal owner for self-information (*p* = 0.001), and recommendations of good information sources (*p* = 0.022) were positively associated with SDM. Looking at the influence of age and gender on the application of SDM, older people and males rated higher. However, the evaluation of the latent factor SDM was based on the self-estimation of the participants. Assuming that younger women were less self-confident, we cannot exclude that young female participants self-evaluated their SDM skills lower than older male participants, although both groups would objectively have the same SDM level. Practitioners who have a positive attitude toward animal owners, who enjoy contact with animal owners and welcome their interest in further (self-)information, show empathic behavior, and have a positive attitude toward SDM are more likely to have better veterinarian-animal owner-relationships.

## Introduction

Successful veterinary practice requires good communication and relationship-building between veterinarians and animal owners (1, 2). The value of communication skills has become an important research focus in human and veterinary medicine (3–6).

In medical practice, relationship-centered care (RCC) has been established to support mutual understanding in medical encounters (7) and improve the satisfaction of patients and clinicians (8, 9). As Frankel already described in 2006 (1), RCC is based on good communication, including asking open-ended questions, listening actively, understanding the animal owners' experiences, sharing information, and showing empathy and respect. In veterinary medicine, the application of the concepts of RCC must be adapted the specific situation: Comparable to pediatric, the patient is a third party that cannot decide upon itself. Veterinarian and animal owner need to take the optimum decision on behalf of the animal (1). Another difference to the situation in human medicine is that – at least in most cases – financial considerations must be taken into account in the process of decision finding (10).

Several working groups have reported that the veterinarian-animal owner relationship is changing, with animal owners increasingly seeking more information and involvement (11–15). The success of veterinary care in terms of compliance and animal health depends on the satisfaction of the animal's owner (16). This satisfaction can be achieved through effective communication, partnership building (17), and meeting the animal owners' expectations (18). Some research groups have studied pet owners' expectations and found that they value high-quality medical care, good communication, and respectful, individualized treatment (10, 18–20).

RCC is a form of care in which animal owners and veterinarians are aware of their mutual relationship (21). This includes respecting the emotions and individual personalities of both parties. Thus, RCC can satisfy the needs of animal owners (16, 22, 23).

Shared decision making (SDM) is thus part of RCC and invites the animal owner to be equally involved in SDM related to their animal's treatment and therapy (24). Successful exchange of all relevant information is a prerequisite for SDM, including information on therapy and treatment options, their pros and cons, and the related costs (10, 18, 19). Today, the concept of SDM is acknowledged as an important part of medical/veterinary care situations and has been shown to improve the therapeutic success and satisfaction of pet owners and veterinarians (25, 26). But, SDM does not necessarily place the responsibility for the decision on the animal owner. In a successful SDM, decision making is genuinely shared between the veterinarian and the animal owner, while at the same time, the amount of decision making the animal owner is able or willing to take, is respected (25). The application of SDM is challenging when veterinarians and animal owner have different needs, and this might become even more challenging, if the differences are due to animal welfare reasons (27).

Several working groups have developed questionnaires to examine the application of SDM in veterinary and human medical research (26, 28, 29).

The rise of animal owners seeking self-information from the internet has made new sources of information accessible to the general public and has resulted in a number of new challenges in the veterinarian-animal owner relationship (11, 12, 15, 30). In contrast to more traditional self-information sources such as friends and family, literature, and other experts, the quality of information from the internet or social media is more varied (31, 32). Therefore, the self-information of animal owners can be positive (animal owner is better prepared and can better participate in SDM) or negative (correction of incorrect information can be time-consuming and result in less trust between the animal owner and veterinarian).

In Germany, the veterinary profession has become increasingly more dominated by females. Certain aspects of RCC and SDM, such as empathy and active listening, are more commonly displayed by females than males (24, 33, 34). But, in general, little is known about the influence of demographic variables like gender and age on RCC and SDM concepts and such knowledge would help to develop more specific trainings.

The aims of this study were to investigate the role of the practice characteristics (e.g., location, animal species), veterinarian characteristics (e.g., age, gender, self-estimation, use of the internet), and their attitudes concerning animal owners seeking self-information. We particularly focused on establishing whether there was any association between SDM, age and gender.

## Materials and Methods

### Questionnaire

As Küper and Merle (11) described, the questionnaire was in German language and included questions on relationship-centered veterinary care and veterinarians' expectations regarding animal owners identified from a literature review. Validated questionnaires developed in human medical research were adapted for veterinary medicine (e.g., replace “physician” by “veterinarian”) (28, 29, 35).

Additional questions included the characterization of the veterinary practice, such as the type and size of the practice, main animal species (small animals, horses, cattle, pigs, small rodents), type of location, and the veterinarian's age and gender. Questions were also included on the following:

- the self-estimation of certain professional competencies and their perceived relevance for success
- the frequency and topics of training attended
- perceived risks for the failure of successful therapy
- self-informed animal owners: perceived reasons for self-information, quality and sources of information
- attitude toward self-information and self-informed animal owners
- attitude toward complementary medicine such as physiotherapy and chiropractice

The questions were selected in collaboration with veterinary researchers and practitioners. The questionnaire was validated in a three-step pretest phase, including expert interviews with 5 researchers (2 epidemiologists, 2 small animal clinicians, 1 sociologist) and 2 veterinary practitioners, cognitive pretesting with eight veterinarians, and a standard pretest with 22 participants (36).

The final questionnaire comprised 65 items, most of which were scored on a six-point Likert scale. The aforementioned influencing factors were either categorical (e.g., location) or continuous (e.g., age). The order of the questions was fixed and demographics were recorded at the end of the questionnaire. Following the feedback of the cognitive pretesting we did not expect bias due to the order of the questions. It took about 15 min to fi in the questionnaire.

### Data Collection

The survey was completed by German veterinary practitioners between November 14, 2016, and June 30, 2017. Veterinarians that had worked in a curative veterinary practice or clinic in the last 2 years could participate. The collection, storage, and processing of the data followed the German data protection laws (37). Veterinarians participated voluntarily and provided informed consent actively beforehand. Data collection was anonymous, and no individual-related or other sensitive data were collected. The survey could be terminated at any point. As per local legislation, approval by an ethics committee was not required.

The questionnaire was available online (LimeSurvey, open-source, hosted on university servers) to reach as many veterinarians as possible. The project website (www.fokustiergesundheit.de) with an external link to the survey page was promoted across veterinarians' Facebook groups. The link was shared by the Federal Veterinary Associations, the German Association of Practicing Veterinarians, and the journal “Deutsches Tierärzteblatt.”

### Data Analysis

Data from the LimeSurvey questionnaires were stored in a Microsoft Excel® (version 2016) spreadsheet and statistically analyzed using the Statistical Package for the Social Sciences for Windows (SPSS version 25, IBM Corp., Armonk, N.Y., USA). The figures were created using Microsoft PowerPoint® 2016.

**Figure 1:**
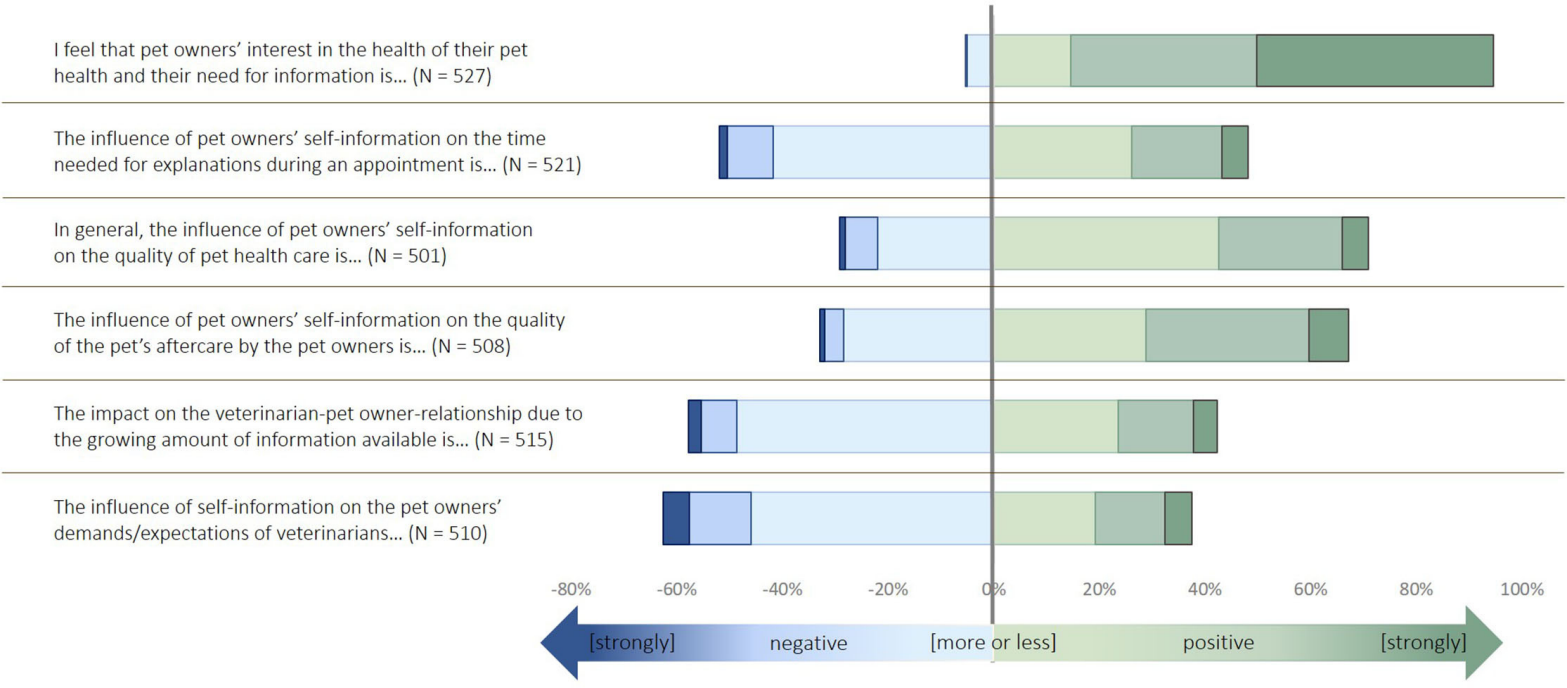
Description of the items used to build the latent factor “shared decision making” in the survey. The items were identified by an exploratory factor analysis and structural equation modeling, as described in Küper and Merle (11).

**Figure 2:**
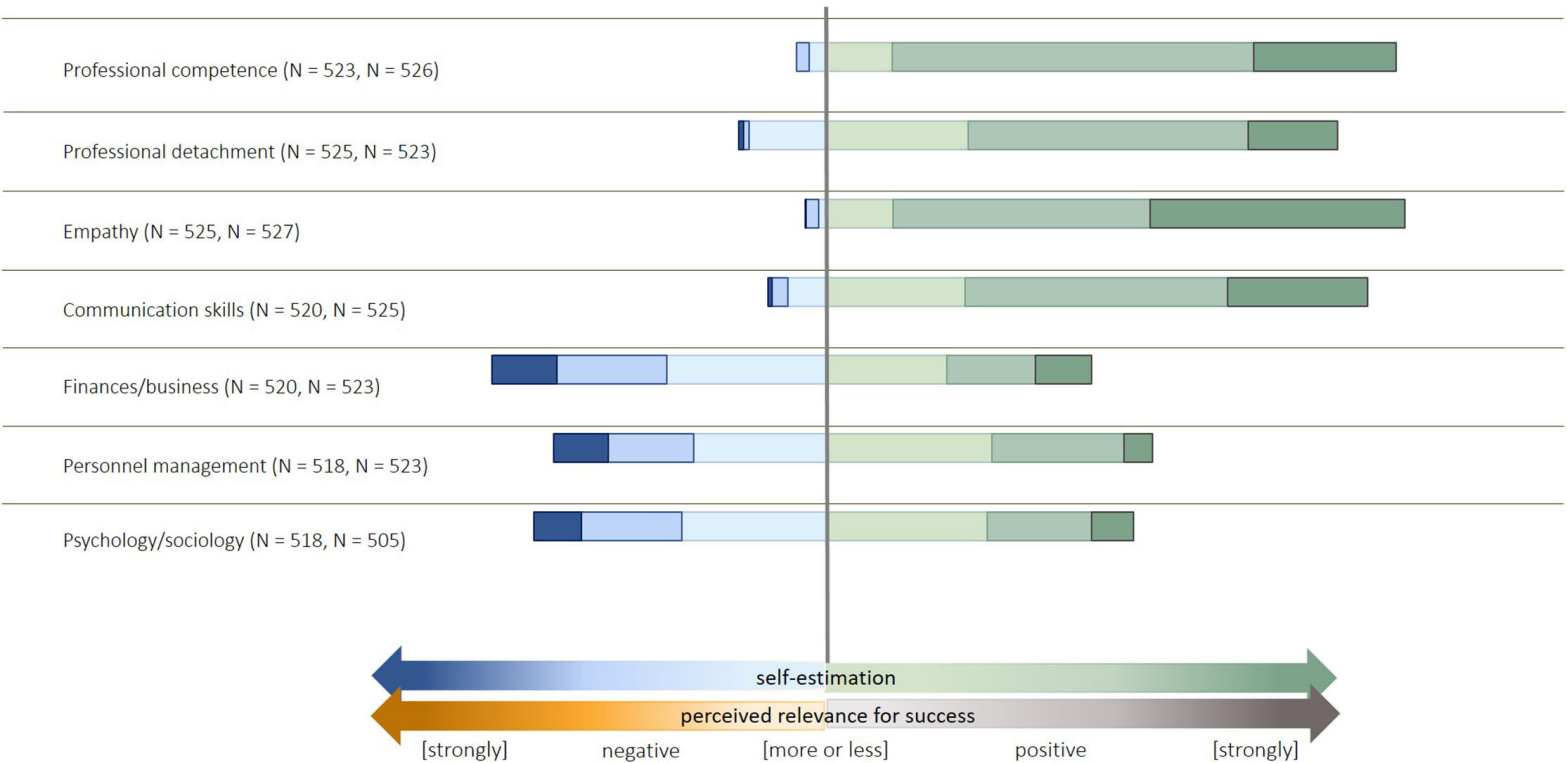
Description of the items used to build the latent factor “expression of empathy” in the survey. The items were identified by an exploratory factor analysis and structural equation modeling, as described in Küper and Merle (11).

**Figure 3:**
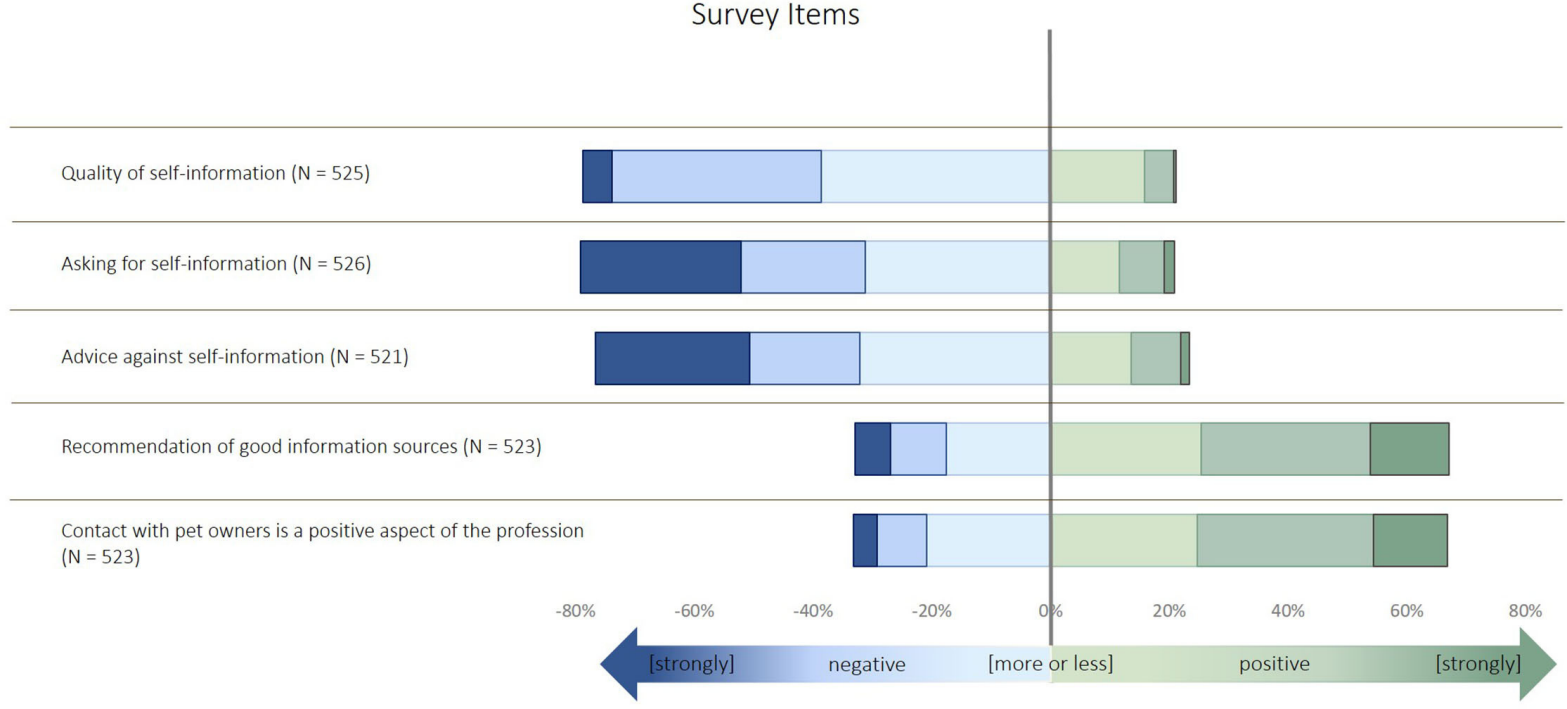
Description of the items used to build the latent factor “perceived impact of animal owners' self-information” in the survey. The items were identified by an exploratory factor analysis and structural equation modeling, as described in Küper and Merle (11).

Küper and Merle (11) published a structural equation model that defines three latent factors that describe a veterinarians' perception of their communication style and ability with pet owners. These are (1) SDM, (2) perceived impact of animal owners seeking self-information, and (3) expression of empathy. These factors were used for further regression analyses. Descriptive results of the latent factors are provided in the results section (Figures 1–3). For further analyses, only participants that indicated to treat at least one of the following species were included: small animals, horses, small rodents, reptiles, or pet birds.

Mann-Whitney-U-test was carried out to investigate differences in age between males and females.

#### Exploratory Factor Analyses

Exploratory factor analyses were conducted so that related questionnaire items could be grouped to the number of variables in the regression model. A promax (oblique) rotation was used to extract the factors. The number of factors was selected following the scree plot (significant break) and eigenvalues (>1) (38).

**Figure 4:**
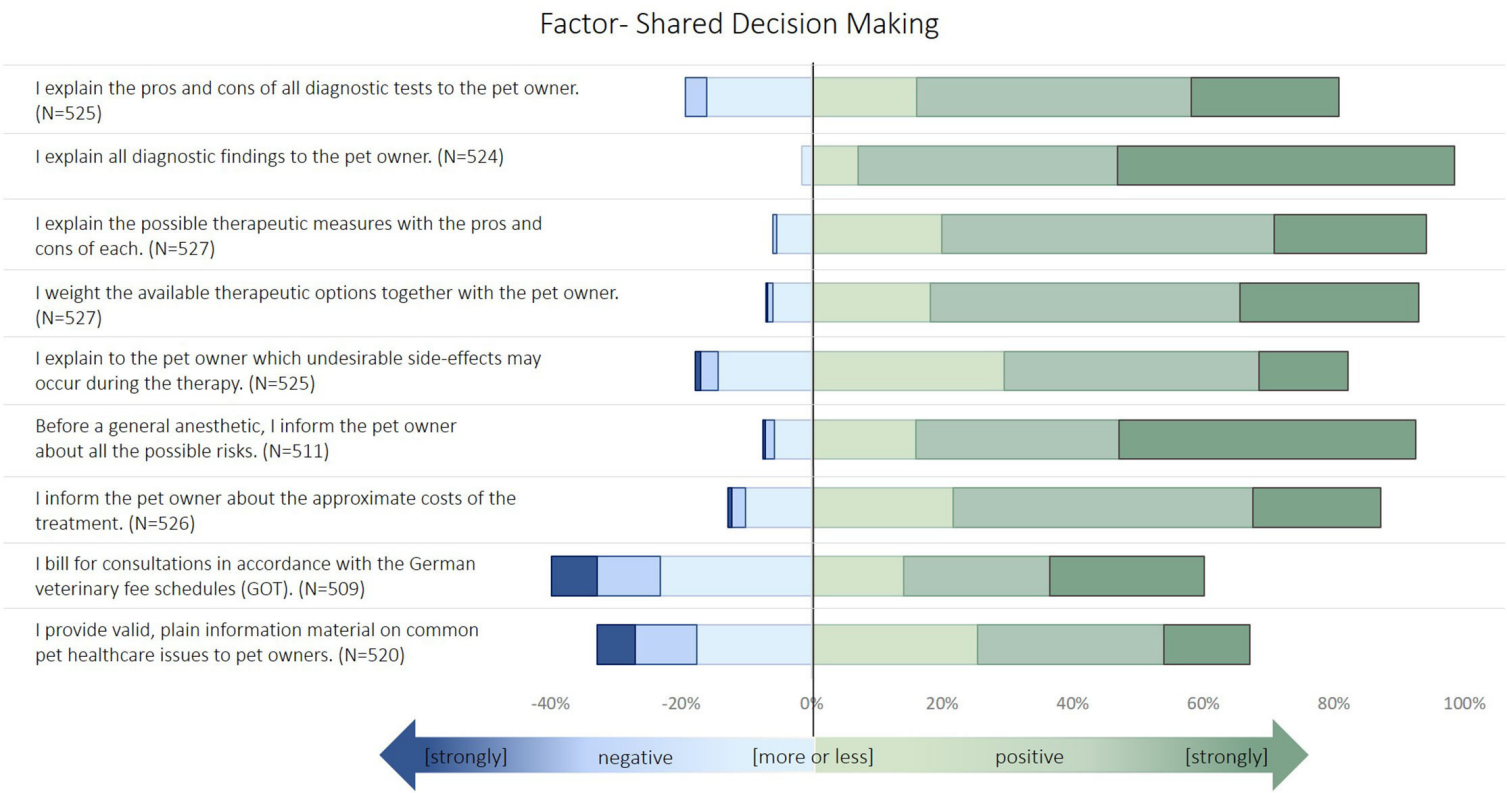
Description of the items used to build the factors relating to self-estimation and perceived relevance for success in the survey.

From the 14 items on self-estimation and the respective relevance for the success of certain professional competencies (Figure 4), three factors were extracted: (1) self-estimation, (2) professional competence and professional detachment, and (3) relevance for success (excl. factor 2). The Kaiser-Meyer-Olkin (KMO) measure was 0.722 [middling according to Kaiser and Rice (39)], and all KMO-measures were > 0.5. Although four factors had eigenvalues above 1, we decided to take only three factors because no item had the highest loads on factor four. In combination, the three factors explained 51.7% of the variance.

Two factors were defined for the perceived risks of therapy failure (10 items): (1) risk factors associated with veterinarians include a lack of communication ability or time, and (2) risk factors associated with animal owners include nervousness or incorrect administration of drugs. The KMO was 0.682 in the mediocre area, but all of the individual KMO values were above 0.7. Although three factors had eigenvalues above 1, we selected two factors to increase the interpretability.

The 12 items regarding the perceived sources of self-information were classified into three factors: (1) literature, (2) experts, and (3) social media. The KMO was again only 0.663 in the mediocre area, but all of the individual KMO values were above 0.8. Three factors were extracted, which accounted for 41.5% of the total variance.

The last three factors were developed from the 11 items that addressed the reasons why animal owners sought self-information: (1) further questions after the consultation, (2) skepticism against veterinarians, and (3) interest in the topic. The KMO was 0.662, and all of the individual KMO values were above 0.8. All three extracted factors had eigenvalues above 1, and the cumulative variance was 43.6%.

#### Regression Models

We used multivariable general linear regression models with manual backward elimination to investigate the influence of risk factors on the latent factor SDM. The candidate values were selected based on their correlation with the dependent variable (Spearman rank correlation coefficient > |0.1|) and consideration of biological associations. The following variables were included: all of the above-described factors plus “professional experience >15 years,” “self-employed,” “animal species” (small animals, horses), “training in communication,” “perceived quality of self-information,” “asking for need for self-information,” “advice against self-information,” “recommendation of good information sources,” “self-information of animal owners goes along with uncertainty,” “openness to complementary medicine,” “perceived educational level of clients,” “gender,” “age,” and “location of the practice.”

The preliminary correlation analysis (Spearman rank correlation) revealed correlation coefficients >0.5 between experience and age, experience and self-employment, the factors empathy and self-information. Thus, experience and self-information were excluded from the analyses. In the first step, we included all the variables described above. All two-way-interactions were included in the model and removed one by one based on their *p*-values until only statistically significant interactions remained. The results of this full model are displayed in Supplementary Table 1 but are not discussed further in this report. Next, we eliminated variables one by one to maximize the R-squared value. Finally, each excluded variable was included in the model one by one. If the R-squared value increased, the variable was kept in the model. Age and gender were forced into the model. *Post-hoc* pairwise comparisons of the categorical variables were adjusted using the Bonferroni method. Model diagnostics included normality and homoscedasticity of the residuals. The level of significance was set at 0.05.

## Results

### Description of the Questionnaire Items

In total, 527 people completed the survey, and 466 observations were included in the regression model (exclusion of 61 observations due to missing values or no treatment of pet animals or horses). The mean age of the veterinarians surveyed was 43.2 ± 10.6 years. The 370 female participants (median 39.0 years) were significantly younger than males (*n* = 96, median 52.0 years; *p* < 0.001, Mann-Whitney-U-test).

The variables used to build the factors are displayed in Figures 1–4 and Tables 1–**3**. The values of the factors were dimensionless results from the exploratory factor analysis with a mean of zero and standard deviation of one.

**Table 1:** Associations between the questionnaire items/factors and age and gender.

	**Independent variables**			
	**Age**		Gender^**^	
**Dependent variable**	**Regression coefficient**	***p*-value**	**Regression coefficient**	***p*-value**
SE^*^ professional competence	0.018	<0.001	0.146	0.115
SE professional detachment	0.003	0.557	0.217	0.061
SE empathy	0.008	0.036	−0.262	0.012
SE communication	0.011	0.017	−0.01	0.932
SE business/finances	0.049	<0.001	0.613	<0.001
SE personnel management	0.057	<0.001	0.249	0.081
SE psychology/sociology	0.045	<0.001	0.008	0.961
Factor self-estimation	0.035	<0.001	0.084	0.399
Factor professional competence and professional detachment	0.002	0.638	−0.097	0.374
Factor relevance for success	−0.004	0.253	0.13	0.147
Factor veterinarian as risk factor for therapy failure	−0.005	0.207	−0.123	0.204
Factor animal owner as risk factor for therapy failure	−0.001	0.791	−0.087	0.348
Contact with animal owners a positive aspect of profession	0.022	<0.001	−0.295	0.053
Factor literature as information source	−0.004	0.264	0.362	<0.001
Factor experts as information source	−0.001	0.768	−0.234	0.009
Factor social media as information source	−0.005	0.038	−0.138	0.077
Perceived quality of information	0.007	0.1212	0.014	0.903
Factor open questions as reason for self-information	−0.009	0.014	0.131	0.165
Factor skepticism as reason for self-information	−0.014	<0.001	−0.006	0.949
Factor interest in animal health as reason for self-information	0.009	0.006	−0.016	0.845

*Results of a survey of 527 German veterinarians*.

* *SE: self-estimation*.

** *Gender: Females are the reference group*.

Most of the veterinarians indicated to follow the concepts of SDM (Figure 1). More than 20% of participants disagreed about billing customs and providing information material. The majority of participants agreed or strongly agreed with the selected expressions of empathy (Figure 2). The variables related to the perceived impact of self-information were rated more diversely (Figure 3). Animal owners' interest in animal health issues was assessed as positive by most participants, but all other items were rated as negative by 30–60% of the participants.

The comparison of perceived self-estimation and perceived relevance for success shows that most of the items were valued in a positive way. Self-estimation was significantly higher in older participants (*p* < 0.001) but not significantly different between males and females (*p* = 0.399, general linear regression, Table 1). Some of the single items concerning self-estimation were also significant such as professional competence (*p* < 0.001), empathy (*p* = 0.038), communication (*p* = 0.017), business/finances (*p* < 0.001), and personnel management (*p* < 0.001). Moreover, psychology/sociology (*p* < 0.001) was rated significantly higher by older people. An additional gender effect could only be observed for the self-estimation of empathy (*p* = 0.012, females rated higher) and business/finances (*p* < 0.001, males rated higher).

The risk for the failure of successful therapy ranged from 26 to 83% (Table 2). Most often, veterinarians assumed that the prescribed drugs or the therapeutic measures were not administered correctly. In contrast, not making an agreement on how to proceed further and a lack of time were selected only by 26 and 30% of the participants, respectively.

**Table 2:** Frequencies of questionnaire items related to the factors “risk factors associated with veterinarians” and “risk factors associated with animal owners” from a survey of 527 German veterinarians.

**Item**	**Count**	**%**
Drugs could not be administered correctly	439	83.30%
Therapeutic measures were not applied correctly	419	79.51%
Drugs were not administered correctly	385	73.06%
Recommendations for how to proceed further were not followed	363	68.88%
Lack of feasibility of the therapy plan in the pet owner's daily life	313	59.39%
Intentional change or ignorance of the therapy plan (pet owner)	290	55.03%
Nervousness of the pet owner during the appointment	269	51.04%
Lack of communication skills (vet)	189	35.86%
Lack of time preventing a comprehensive explanation (vet).	160	30.36%
Recommendations for how to proceed further were not clear.	139	26.38%

The internet and friends were regarded as very important sources of information, while journals, brochures, training courses, books, or publications were not (Table 3). The reasons why animal owners sought further information were also estimated by the veterinarians (Table 4). They did not feel that their own involvement, such as a lack of empathy or time, influenced the decision of owners to seek self-information very often (14 and 13%, respectively). Rather, veterinarians felt that the owners sought self-information due to being concerned about their animal, interest in animal health, and the wish to save money.

**Table 3:** Frequencies of the questionnaire items related to the sources of self-information from a survey of 527 German veterinarians.

**Item**	**Count**	**%**
Internet forum	505	95.83%
Friends	428	81.21%
Internet pages	415	78.75%
Breeder	315	59.77%
Facebook group	310	58.82%
Trainer	297	56.36%
Another vet	161	30.55%
Journals	40	7.59%
Brochures	18	3.42%
Training courses	13	2.47%
Books	11	2.09%
Publications	10	1.90%

**Table 4:** Frequencies of the questionnaire items related to reasons that owners sought self-information from a survey of 527 German veterinarians.

**Item**	**Count**	**%**
Concerned about the animal	327	62.05%
Interest in animal health/diseases	313	59.39%
To save money	307	58.25%
Need for a second opinion	301	57.12%
Lack of trust in conventional medicine	299	56.74%
Preparation of veterinarian consultation	248	47.06%
Lack of trust in veterinarian's competence	195	37.00%
To clarify aspects that remained unclear because the pet owner did not feel comfortable asking the veterinarian	136	25.81%
To understand complex aspects of the treatment or disease better in a written form	90	17.08%
Lack of empathy by the veterinarian	72	13.66%
To clarify aspects that remained unclear due to the limited consultation time	69	13.09%

**Figure 5:**
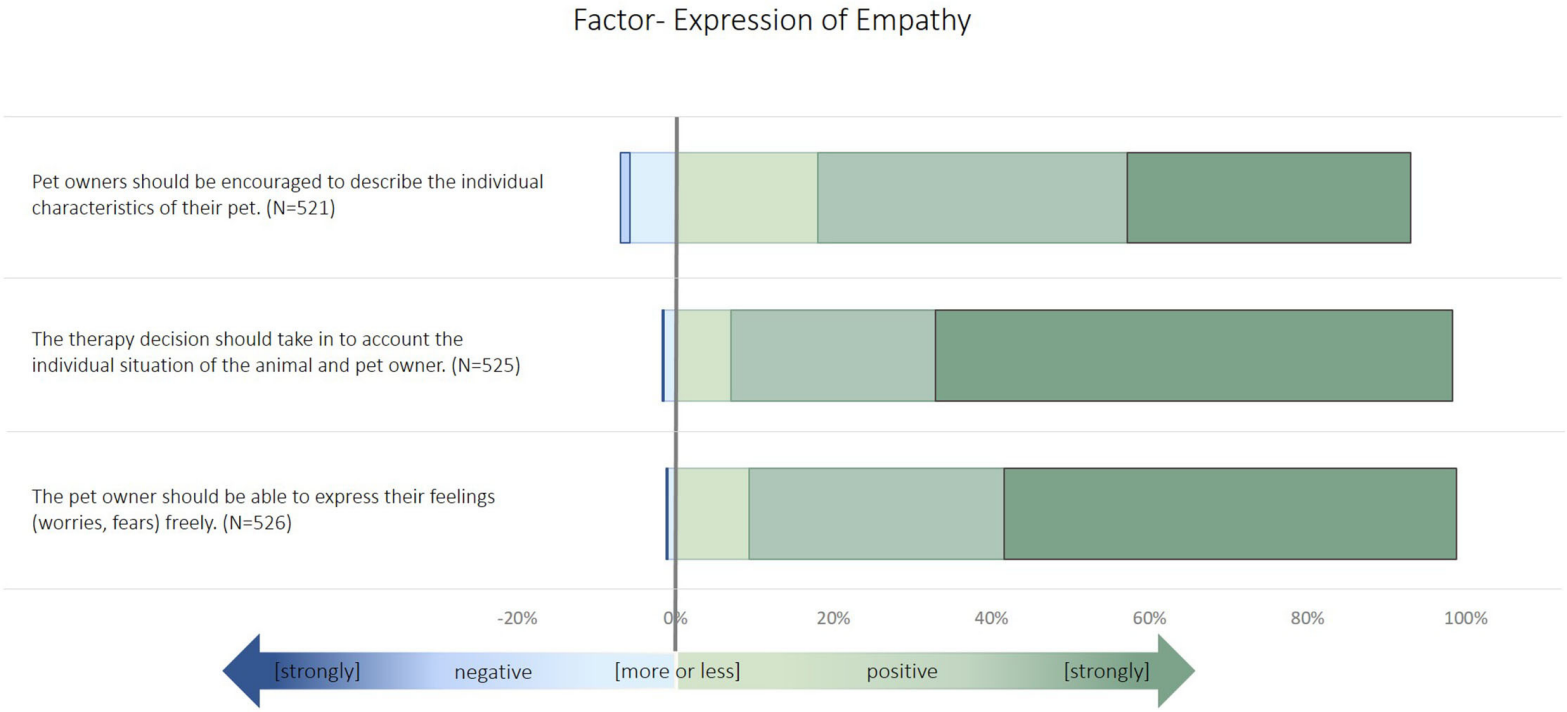
Description of the questionnaire items in the survey on shared decision making taken by German veterinarians.

The descriptions of the remaining variables are displayed in Figure 5. The quality of self-information was regarded as negative by the participants. Although most participants did not ask for self-information, they did not advise against self-information but recommended good information sources. Taking age into account in the model, gender was no longer statistically significant in terms of social media (*p* = 0.077), but age was (*p* = 0.038, general linear model, Table 1): the older participants were, the less the factor social media was perceived as source for self-information.

The contact with animal owners as a positive aspect of the profession was rated significantly higher in older people (*p* < 0.001) and in females (although this was not formally significant: *p* = 0.053, general linear model). The older participants were, the less often they assumed that open questions (*p* = 0.014) or skepticism (*p* < 0.001) were the reasons for animal owners seeking self-information. Rather, older people assumed that animal owners sought self-information due to pure interest (*p* = 0.006). No significant gender effects were observed. Females rated the frequency of literature as an information source lower (*p* < 0.001), and the respective impact of experts higher (*p* = 0.009) than males.

### Multivariable Analysis of SDM

The results of the multivariable general linear regression model are displayed in Table 5. The location of the practice was statistically significant. The metropolis had the highest values, followed by the commuter belt, cities, and rural areas. Significant differences were only observed between the metropolis and rural areas (*p* = 0.005 in Bonferroni *post-hoc* comparisons). Veterinarians who assumed factors associated with veterinarians were important as the reason for therapy failure had significantly higher SDM values. If the respective factors associated with animal owners were regarded as important, SDM was significantly lower. A negative association with SDM was also detected for the perceived quality of self-information, and the factor “skepticism as the reason for self-information.” Those veterinarians who advised against self-information and those who believed that self-information goes along with uncertainty had low SDM values as well. If the literature was presumed to be an information source and interest as the reason for self-information, then the SDM values were significantly high. Questions for the need for self-information, and recommendations for good information sources were positively associated with SDM. A total of 38.5% of the overall variance could be explained by the model (*R*^2^ 0.385).

**Table 5:** Results of the final multivariable linear regression model with SDM as a dependent factor after backward selection.

**Dependent variable: factor- shared decision making**		***n***	**Regression parameter**	**Standard error**	***t*-value**	***p*-value**	**95% confidence interval**	
							**Lower Bound**	**Upper Bound**
	Intercept		0.735	0.310	2.369	0.018	0.125	1.344
Localization of practice	Rural area	145	−0.129	0.095	−1.363	0.173	−0.316	0.057
	City	121	−0.023	0.100	−0.230	0.818	−0.220	0.174
	Metropolis	101	0.197	0.105	1.878	0.061	−0.009	0.404
	Commuter belt	99	0.000	.	.	0.010	.	.
Specialized in	Small animals	421	0.151	0.134	1.128	0.260	−0.112	0.414
	Horses	94	−0.157	0.103	−1.520	0.129	−0.361	0.046
	Factor: expression of empathy	466	0.064	0.039	1.617	0.107	−0.014	0.141
	Factor: professional competence and professional detachment	466	−0.070	0.037	−1.908	0.057	−0.142	0.002
	Factor: relevance for success (excl. factor 2)	466	0.062	0.045	1.379	0.168	−0.027	0.152
	Factor: risk factors associated with veterinarians	466	0.132	0.043	3.052	0.002	0.047	0.217
	Factor: risk factors associated with animal owners	466	−0.142	0.044	−3.189	0.002	−0.229	−0.054
	Perceived quality of self-information	466	−0.213	0.039	−5.432	<0.001	−0.290	−0.136
	Factor: literature as information source	466	0.129	0.054	2.365	0.018	0.022	0.236
	Factor: skepticism as reason for self–information	466	−0.185	0.054	−3.409	0.001	−0.292	−0.079
	Factor: interest as reason for self-information	466	0.218	0.059	3.675	<0.001	0.101	0.334
	Asking for need for self-information	466	0.091	0.029	3.206	0.001	0.035	0.147
	Advice against self-information	466	−0.075	0.027	−2.757	0.006	−0.128	−0.021
	Recommendation of good information sources	466	0.062	0.027	2.296	0.022	0.009	0.114
	Self-information of animal owners goes along with uncertainty	466	−0.113	0.035	−3.203	0.001	−0.182	−0.044
Gender	Male	96	0.114	0.093	1.233	0.218	−0.068	0.296
	Female	370	0.000	.	.	.	.	.
	Age	466	0.008	0.004	2.100	0.036	0.000	0.015

*Adjusted R-squared: 0.385*.

Older veterinarians and those who expressed themselves as having high levels of empathy tended to have higher SDM values, although the latter factor was not significant. Male participants had slightly higher SDM values than females. The application of SDM was lower in practices that specialized in horses and higher in those that specialized in small animals. Veterinarians that rated professional competence and detachment high, had lower levels of SDM, while those that rated other factors for success high, had higher levels of SDM.

## Discussion

Many factors were associated with the latent factor SDM. The R-squared values show that a substantial part of the veterinarians' attitude toward SDM can be explained by the factors in the final model.

We decided to perform a factor analysis. The item sets of one topic were condensed to two or three factors each. This helped us avoid multicollinearity between independent variables and to reduce the number of variables in the model. We could interpret each extracted factor in a meaningful way, which allowed us to test hypotheses in a more general way. Although the model quality of the factor analyses was restricted (all of the KMOs were below 0.8 and explained total variances below 50%), in our view, the benefits outweighed this weakness. Nevertheless, the interpretation of these factors must be made carefully.

The data cannot be regarded as representative because of our convenience sampling strategy used. This means that people participated voluntarily, which typically leads to selection bias. Additionally, the survey was available only online, which again means that only internet users were included. Thus, the study results do not reflect those of all German veterinarians and care should be taken before generalization. It must also be taken into account that the study results only reflect the attitudes and perceptions of the veterinarians. In the corresponding survey of animal owners, we intentionally invited participants independently from their veterinarians to avoid bias due to social desirability. Therefore, we don't know if the animal owners would agree on the veterinarians' perceptions and SDM application.

The R-squared of 38.5% shows that although many potential influence factors were covered by the model, there are still a lot of unexplained influence factors that need to be detected in future investigations. This is characteristic for complex multifactorial questions that often arise in studies with sociological and/or psychological content.

However, there were no fundamental differences between the participants' demographic characteristics and the official statistics of the Federal Chamber of Veterinarians (40). About 66% of all the practitioners were female, while in our study, 79% of the participants were female. This might be a source of bias. Furthermore, the participants of the current study tended to be younger, which may be due to the described selection bias. Among participants and all veterinarians, more males were present in older age groups than younger age groups. This is in agreement with the high percentage of female veterinary students (85.6% in German universities in 2019) (40, 41).

There is a shift in gender distribution that entails a change in the veterinary profession. A recent study among young, employed practitioners showed that a good working atmosphere and sufficient time for family were significantly more important for females than males, while salary was more important for males (33, 42). In general, females tended to be less satisfied with the current working situation than males. The increasing number of female practitioners are potential drivers for a change in the veterinarian-animal owner relationship with an increasing focus on “soft skills” such as communication, empathy, and SDM characteristics (43).

In our study, no significant difference was found between males and females concerning the application of SDM. However, we found significant associations with gender for some factors related to personal characteristics and self-estimation. Females tended to rate their level of empathy higher, but their competence in business and financial aspects lower. Higher effects were also observed concerning the positive assessment of contact with animal owners and the estimation of experts as information sources. All these findings can only be regarded as rough indicators of the role of gender in SDM. We did not include further confounding factors except of age and gender in these general linear models. We also did not account for multiple testing, and the normality of residuals was not always given due to the ordinal scaling of the parameters. In conclusion, the reason why gender was not significant in the final model, might be that this gender effect was covered by the effects of self-estimation. However, our findings are consistent with many other studies (34, 41, 44–46).

According to the results of other studies (43), older people had higher self-estimation and self-confidence. Older participants rated contact with animal owners more positively and did not presume that the need for further information might be due to their own behavior. Interestingly, older people also had higher SDM values than younger people. Regarding the reliability of the results, the same applies as described above for gender. Professional experience needed to be eliminated from the model. However, we assume that higher self-confidence and self-estimation are due to professional experience. In addition, older age might be associated with higher imperturbability in some people, which could also explain why older participants described themselves as being more open to self-informed animal owners.

Looking at the influence of age and gender on the application of SDM, the hypothesis that younger and female participants would have higher values could not be proven. In fact, the opposite was found, with older people and males rating higher. In addition to the above discussed associations with some factors, another point may also play a role. The evaluation of the latent factor SDM was based on the self-estimation of the participants. Those who were less self-confident tended to evaluate their behavior more critically than those with a higher self-confidence level. Therefore, we cannot exclude the fact that young female participants self-evaluated their SDM skills lower than older male participants, although both groups would objectively have the same SDM level.

Concerning SDM, the clarity of the results was surprising. All factors that represent a positive attitude toward animal owners were positively associated with SDM (e.g., open questions and interest as the reason for self-information regarding contact with animal owners positive). Factors that reflect negative experiences with self-informed animal owners were negatively associated with SDM, for example, presuming that animal owners were the source of therapy failure, assessing the quality of self-information as low, presuming social media to be the information source, and skepticism as the reason for self-information. All these factors correspond to the personal attitudes and experiences of veterinarians. Potential bias may occur since the results only display the self-estimation of the participating veterinarians and not the assessment of their clients. Gender and age might contribute to these personal characteristics.

Animal owners have different demands on the veterinarian-animal owner relationship depending on the location of the practice and animal species. In rural areas, animal owners' need for further information was lower than in towns (47), while animal owners in larger towns expected the application of SDM. We found a clear difference in the veterinarians' assumptions concerning animal owners' attitudes toward SDM between metropolises with related commuter belts and smaller cities or rural areas.

The study results made clear that many animal owners need self-information and that the use of the internet or social media to find information is high (11). This can have negative consequences for practitioners because the quality of the information varies, and correcting misinformed animal owners can be very time-consuming (32, 35). Thus, some veterinarians negatively regard animal owners seeking self-information. This corresponds with findings in human medicine, with some physicians viewing self-informed patients negatively (48).

Studies in human medicine have shown that addressing health information from the internet during medical appointments can improve communication (48, 49). In addition, most patients or animal owners do not mistrust their veterinarians but simply want to have some background information. Thus, often they do not discuss their internet findings with the doctor (or veterinarian) because they do not want to challenge the clinician (12, 50). Veterinarians can motivate animal owners to inform themselves, provide their own (written) information, and recommend good information sources. They can ask what the animal owner already knows and provide further information. These measures can help to build trust and strengthen a good relationship. In addition, they pave the way to shared decision making (6).

Interestingly, so far, only a few studies have focused on physician's communication preferences (51), and no study has been published that highlights the attitude of veterinarians toward SDM. Although patients prefer SDM as a communication model, SDM methods are not fully applied in general practice (20, 52).

## Conclusions and Outlook

In conclusion, we found that many factors are associated with SDM. Practitioners who have a positive attitude toward animal owners, who enjoy contact with animal owners and welcome their interest in further (self-)information, show empathic behavior, and have a positive attitude toward SDM are more likely to have better veterinarian-animal owner-relationships. To improve veterinarians' ability to apply SDM and RCC in daily practice, courses should be offered to students and post-graduates (4). The skills of SDM and RCC will become increasingly important due to the changing views of veterinarians and animal owners.

## Data Availability Statement

The raw data supporting the conclusions of this article will be made available by the authors, without undue reservation.

## Author Contributions

AK conceived and designed the study, developed the theoretical framework, implemented it into the preliminary model and questionnaire, and revised the paper. Statistical preliminary considerations were done by AK and RM. RM conducted statistical analyses and drafted the manuscript. All authors contributed to the article and approved the submitted version.

## Funding

Open Access Funding provided by the Freie Universität Berlin.

## Conflict of Interest

AK was temporarily employed in a Start-up business with interest in Digital Animal Health Care (vetevo GmbH) that potentially could have been interested in the study results. The employment relationship started almost 1 year after the start of the research project and ended before publications were completed. The company was not involved in any steps of study design, data collection or evaluation, and no data or findings were provided to the company. Potential conflicts were prevented by obligation toward the privacy statements as well as the policies of good scientific work of the Institute for Veterinary Epidemiology and Biostatistics. The remaining author declares that the research was conducted in the absence of any commercial or financial relationships that could be construed as a potential conflict of interest.

## Publisher's Note

All claims expressed in this article are solely those of the authors and do not necessarily represent those of their affiliated organizations, or those of the publisher, the editors and the reviewers. Any product that may be evaluated in this article, or claim that may be made by its manufacturer, is not guaranteed or endorsed by the publisher.
