# Supplementary material for: Attitude of Veterinarians Toward Self-Informed Animal Owners Affects Shared Decision Making
Source: Front Vet Sci. 2021 Oct 20;8:692452. doi: 10.3389/fvets.2021.692452 (PMC8564114; doi:10.3389/fvets.2021.692452)
Supplement: Supplementary file 1 [file Table_1.DOCX]

| **Supplementary Table 1. Results of the multivariable linear regression model with SDM as a dependent factor. The full model includes all predefined influencing factors. Adjusted R-squared: 0.378.** | | | | | | | | |
| --- | --- | --- | --- | --- | --- | --- | --- | --- |
| Dependent Variable:  Factor- Shared Decision Making | | n | Regression parameter | Standard Error | t-value | p-value | 95% Confidence Interval | |
|  |  |  |  |  |  |  | Lower Bound | Upper Bound |
|  | Intercept |  | 0,646 | 0,351 | 1,839 | 0,067 | -0,044 | 1,336 |
| Localization of practice | rural area | 145 | -0,122 | 0,098 | -1,244 | 0,214 | -0,314 | 0,071 |
|  | city | 119 | -0,016 | 0,102 | -0,156 | 0,876 | -0,217 | 0,185 |
|  | metropolis | 100 | 0,207 | 0,108 | 1,926 | 0,055 | -0,004 | 0,419 |
|  | commuter belt | 98 | 0,000 | . | . | 0,013 | . | . |
| Specialized in | small animals | 417 | 0,166 | 0,137 | 1,212 | 0,226 | -0,103 | 0,435 |
|  | horses | 94 | -0,130 | 0,107 | -1,210 | 0,227 | -0,341 | 0,081 |
|  | self-employed | 264 | 0,049 | 0,093 | 0,525 | 0,600 | -0,133 | 0,231 |
|  | factor: expression of empathy | 462 | 0,059 | 0,041 | 1,442 | 0,150 | -0,021 | 0,140 |
|  | factor: self-estimation | 462 | 0,005 | 0,048 | 0,112 | 0,911 | -0,088 | 0,099 |
|  | factor: professional competence and processional detachment | 462 | -0,078 | 0,039 | -1,974 | 0,049 | -0,155 | 0,000 |
|  | factor: relevance for success (excl. factor 2) | 462 | 0,064 | 0,046 | 1,379 | 0,169 | -0,027 | 0,155 |
|  | factor: risk factors associated with veterinarians | 462 | 0,125 | 0,046 | 2,735 | 0,006 | 0,035 | 0,214 |
|  | factor: risk factors associated with animal owners | 462 | -0,141 | 0,047 | -2,975 | 0,003 | -0,234 | -0,048 |
|  | perceived quality of self-information | 462 | -0,200 | 0,041 | -4,888 | <0.001 | -0,280 | -0,119 |
|  | factor: literature as information source | 462 | 0,121 | 0,056 | 2,167 | 0,031 | 0,011 | 0,232 |
|  | factor: experts as information source | 462 | 0,064 | 0,064 | 1,013 | 0,311 | -0,060 | 0,189 |
|  | factor: social media as information source | 462 | -0,114 | 0,080 | -1,425 | 0,155 | -0,272 | 0,043 |
|  | factor: open questions as reason for self-information | 462 | 0,053 | 0,056 | 0,944 | 0,346 | -0,057 | 0,162 |
|  | factor: scepticism as reason for self-information | 462 | -0,195 | 0,062 | -3,140 | 0,002 | -0,317 | -0,073 |
|  | factor: interest as reason for self-information | 462 | 0,184 | 0,070 | 2,630 | 0,009 | 0,047 | 0,322 |
|  | training in communication | 462 | 0,007 | 0,077 | 0,088 | 0,930 | -0,144 | 0,157 |
|  | asking for need for self-information | 462 | 0,097 | 0,030 | 3,275 | 0,001 | 0,039 | 0,155 |
|  | advice against self-information | 462 | -0,073 | 0,027 | -2,668 | 0,008 | -0,127 | -0,019 |
|  | recommendation of good information sources | 462 | 0,055 | 0,027 | 2,023 | 0,044 | 0,002 | 0,109 |
|  | self-information of animal owners goes along with uncertainty | 462 | -0,114 | 0,036 | -3,183 | 0,002 | -0,184 | -0,044 |
|  | openness to complementary medicine | 462 | 0,026 | 0,025 | 1,030 | 0,303 | -0,023 | 0,075 |
|  | perceived educational level of clients | 462 | -0,001 | 0,037 | -0,023 | 0,981 | -0,073 | 0,071 |
| Gender | male | 93 | 0,099 | 0,097 | 1,020 | 0,308 | -0,092 | 0,290 |
|  | female | 369 | 0,000 | . | . | . | . | . |
|  | age | 462 | 0,005 | 0,005 | 0,980 | 0,328 | -0,005 | 0,014 |
